# From mammals back to birds: Host-switch of the acanthocephalan *Corynosoma australe* from pinnipeds to the Magellanic penguin *Spheniscus magellanicus*

**DOI:** 10.1371/journal.pone.0183809

**Published:** 2017-10-05

**Authors:** Jesús Servando Hernández-Orts, Martha Brandão, Simona Georgieva, Juan Antonio Raga, Enrique Alberto Crespo, José Luis Luque, Francisco Javier Aznar

**Affiliations:** 1 Centro de Investigación Aplicada y Transferencia Tecnológica en Recursos Marinos Almirante Storni (CIMAS–CCT CONICET–CENPAT) y Escuela Superior de Ciencias Marinas (ESCiMar), Universidad Nacional del Comahue, San Antonio Oeste, Río Negro, Argentina; 2 Departamento de Parasitologia Animal, Universidade Federal Rural do Rio de Janeiro, Seropédica, Rio de Janeiro, Brazil; 3 Institute of Parasitology, Biology Centre, Czech Academy of Sciences, České Budějovice, Czech Republic; 4 Instituto Cavanilles de Biodiversidad y Biología Evolutiva, Parque Científico, Universidad de Valencia, Paterna, Valencia, Spain; 5 Centro para el Estudio de Sistemas Marinos CESIMAR, CENPAT-CONICET, Puerto Madryn, Chubut, Argentina; 6 Universidad Nacional de la Patagonia San Juan Bosco UNPSJB, Sede Puerto Madryn, Puerto Madryn, Chubut, Argentina; Institute of Tropical Medicine, JAPAN

## Abstract

Trophically-transmitted parasites are regularly exposed to potential new hosts through food web interactions. Successful colonization, or switching, to novel hosts, occur readily when ‘donor’ and ‘target’ hosts are phylogenetically related, whereas switching between distantly related hosts is rare and may result from stochastic factors (i.e. rare favourable mutations). This study investigates a host-switching event between a marine acanthocephalan specific to pinnipeds that is apparently able to reproduce in Magellanic penguins *Spheniscus magellanicus* from Brazil. Detailed analysis of morphological and morphometrical data from acanthocephalans from penguins indicates that they belong to *Corynosoma australe* Johnston, 1937. Partial fragments of the 28S rRNA and mitochondrial *cox*1 genes were amplified from isolates from penguins and two pinniped species (i.e. South American sea lion *Otaria flavescens* and South American fur seal *Arctocephalus australis*) to confirm this identification. Infection parameters clearly differ between penguins and the two pinniped species, which were significantly lower in *S*. *magellanicus*. The sex ratio of *C*. *australe* also differed between penguins and pinnipeds; in *S*. *magellanicus* was strongly biased against males, while in pinnipeds it was close to 1:1. Females of *C*. *australe* from *O*. *flavescens* were smaller than those from *S*. *magellanicus* and *A*. *australis*. However, fecundity (i.e. the proportion of fully developed eggs) was lower and more variable in females collected from *S*. *magellanicus*. At first glance, the occurrence of reproductive individuals of *C*. *australe* in Magellanic penguins could be interpreted as an adaptive colonization of a novel avian host through favourable mutations. However, it could also be considered, perhaps more likely, as an example of ecological fitting through the use of a plesimorphic (host) resource, since the ancestors of *Corynosoma* infected aquatic birds.

## Introduction

Most parasites are considered resource specialists, exploiting only a small number of the potential available hosts in the habitat [1]. However, although host specificity should to be promoted for a variety of reasons (e.g. [2–4]), there are numerous instances of host range expansions and eventual shifts (i.e. host switching) onto phylogenetically unrelated hosts over time [5,6]. In purely descriptive terms, such host-switching events can easily accounted for by two factors, namely, the ability of the parasite to readily contact the new host (either passively or actively), and to survive and reproduce in (on) it [7–9]. In functional terms, successful host shifts are usually explained as a positive adaptive balance between the risks of dispersal and transmission, and the realized fitness in novel hosts [10,11].

It is more difficult to identify, however, the actual mechanisms whereby host range expansions, or shifts, will occur. Specific host-switching events are inherently stochastic, i.e. it cannot be predicted [9], but they are more likely when ‘donor’ and ‘target’ hosts are more closely related, presumably because the physiological accommodation to the latter is easier [12,13]. However, the extent to which such accommodation primarily results from selection of new mutations *vs* existing genetic variation or phenotypic plasticity is an open question. Several authors [1,14–16] have argued that ecological fitting [17] could play a key role in this type of processes. Accordingly, parasites would survive and persist in novel hosts by means of characters that they already possess due to past interactions, i.e., no new mutations are required [1,15]. Ecological fitting would convincingly explain how host-parasite associations become diversified without the concurrence of cospeciation or rare ‘lucky’ mutations [1].

Host shifts are difficult to document and analyse except as a *fait accompli*. However, in this paper we report on a presumably ongoing host-switching event between an acanthocephalan specific to pinnipeds that is apparently able to reproduce, to a lesser extent, in sympatric marine birds. Available evidence suggest that this could represent a case of phylogenetic conservatism, i.e. ecological fitting *via* the use of a plesiomorphic resource [16].

*Corynosoma* Lühe, 1909 (Polymorphidae) is one of the most speciose genus of acanthocephalans, including more than 40 species [18,19]. Species of *Corynosoma* typically reproduce in aquatic mammals or birds [18], but one species, *C*. *seropedicus* Machado Filho, 1970, was described from dogs [20], which are nonetheless considered as accidental hosts [21]. Although the complete life-cycles of most species of *Corynosoma* is unknown, available evidence from several species indicates that benthic amphipods act as intermediate hosts for the larval stages, i.e. the acanthella and the cystacanth (e.g. [22,23]). However, cystacanths of many species of *Corynosoma* have been reported worldwide in a wide array of teleost species. Fish are infected by feeding on infected amphipods and may serve as paratenic hosts that bridge the trophic gap between intermediate and definitive hosts (e.g. [22,24]). Interestingly, cystacanths can circulate widely through the trophic web *via* fish-to-fish transmission [25].

Trophic guilds facilitate contact of species of *Corynosoma* with a number of fish-eating mammals and birds (see [26–28]). There is indeed a number of reports of species of *Corynosoma* typical from pinnipeds in cetaceans [29], marine birds [29,30], the sea otter, *Enhydra lutris* (L.) [31], or even fish-eating terrestrial mammals and birds [32,33]. Conversely, pinnipeds have been reported to harbour species of *Corynosoma* typical from cetaceans [28,34,35] or from marine birds [36]. In any event, species of *Corynosoma* exhibit clear patterns of host specificity to their definitive hosts. From a total of 29 species described as adults in natural hosts, 20 species reproduce in pinnipeds, four in cetaceans, three in aquatic birds, one in the sea otter, and one in the Australian water rat, *Hydromys chrysogaster* Geoffroy (see S1 Table). In contrast, reports of adult specimens of *Corynosoma* spp. in mammals and birds other that their typical hosts are exceptional (S1 Table), and can probably be determined by a compatibility filter (*sensu* [7]).

The above patterns of host-parasite specificity rely on a correct diagnosis of parasite taxa. Identification of species of *Corynosoma* has traditionally been based on morphological characters (e.g. [18]), and several species are diagnosed using subtle differences in the number and arrangement of proboscis hooks, body armature, and morphometry (e.g. [34,37–39]). However, the extent to which these subtle differences may represent instances of intraspecific variation is an open question. Conversely, true diversity (and specificity) of helminth taxa could be underestimated if cryptic speciation has occurred (e.g. [40]). Thus, ideally, morphological and molecular data should be combined to facilitate the diagnosis of taxa (see, [39,41]).

*Corynosoma australe* Johnston, 1937 is a common acanthocephalan specific to pinnipeds from the Southern Hemisphere [28]. At present, there is also a number of records of individuals of *C*. *australe* in sympatric cetaceans and some marine birds, but worms have never been observed to mature [28]. However, during a parasitological survey on the Magellanic penguin *Spheniscus magellanicus* (Foster) in Brazil, we frequently detected putative adult specimens of *C*. *australe* in the intestine. This finding challenged the classic tenet that species of *Corynosoma* are specific to either birds or mammals ([42]; see also S1 Table) and prompted us to carry out a more detailed analysis. In this paper, we firstly provide a thorough morphological description of these specimens and compare them with previous descriptions of *C*. *australe*. Secondly, we compare sequences from isolates of *C*. *australe* obtained from two sympatric pinniped species of the south-western Atlantic [43], with those from specimens collected from penguins. Finally, we assess key fitness-related traits of *C*. *australe* in sympatric penguins and pinnipeds. The results shed light on a probable ongoing process of host-switching *via* ecological fitting.

## Materials and methods

### Sample collection

**Fig 1:**
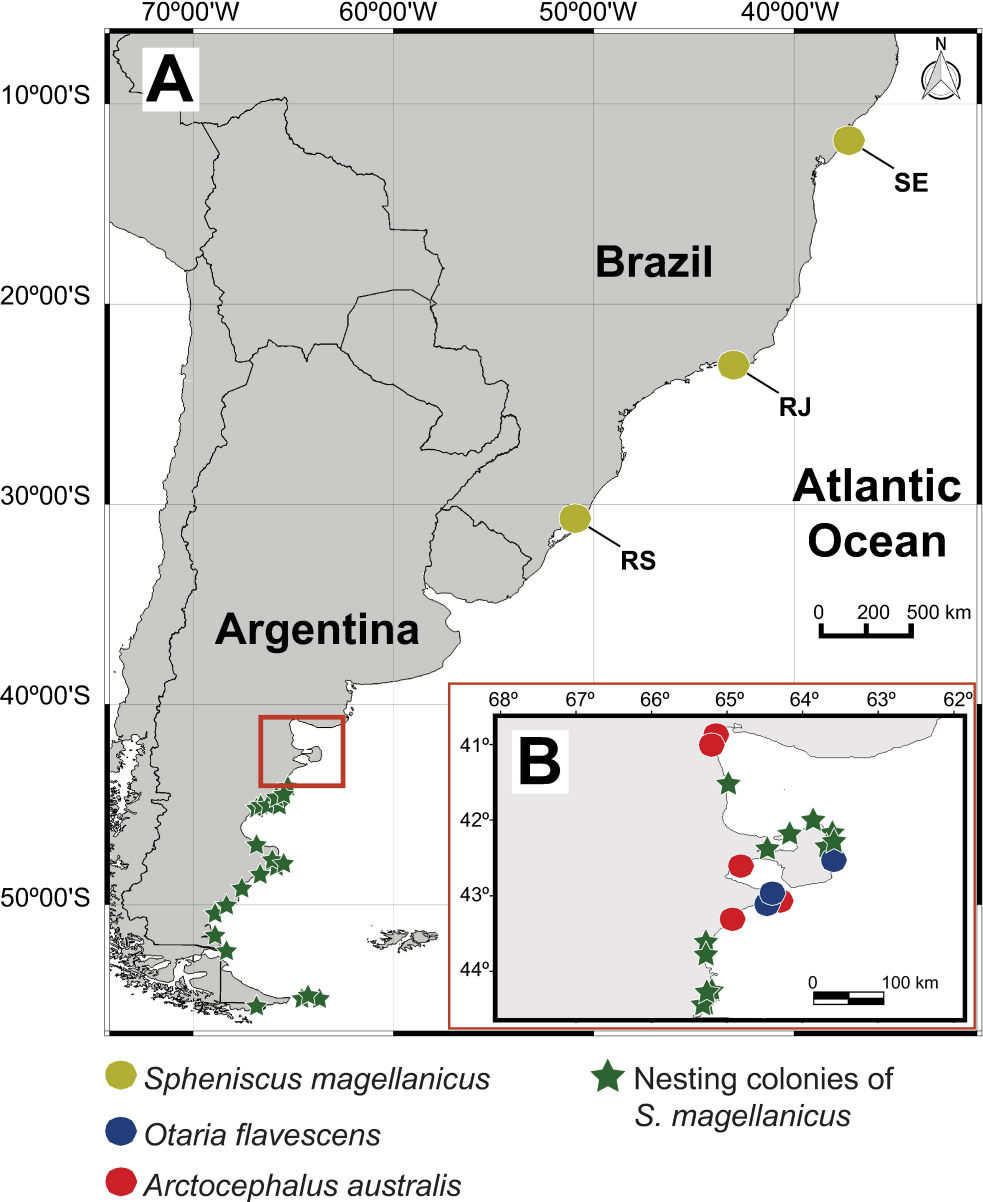
Map of the study area showing the localities where *Spheniscus magellanicus*, *Otaria flavescens* and *Arctocephalus australis* were collected. **A** Southwest Atlantic coast of South America. **B** Detail of the north Patagonian coast of Argentina (red rectangle in Fig 1A). The distribution of the nesting colonies of *Spheniscus magellanicus* in the Atlantic coast of South America follows Schiavini et al. [44]. *Abbreviations*: RJ, Rio de Janeiro; RS, Rio Grande do Sul; SE, Sergipe. The map was made using QGIS v.2.18 (http://www.qgis.org/es/site/). Original copyright [2017].

No ethic statement is required for this study. A total of 87 dead Magellanic penguins were collected on the beaches in the states of Rio de Janeiro (*n* = 71; 55 females, 15 males and 1 individual of undetermined sex), Rio Grande do Sul (*n* = 4; all individuals of undetermined sex) and Sergipe (*n* = 12; all individuals of undetermined sex) in Brazil (Fig 1A), during a peak of penguin mortality in the austral winter and spring of 2008 and 2010. The intestine of each freshly dead bird was removed from the carcass and frozen. After thawing, the intestine was opened and the contents were washed with tap water over a sieve 75 μm mesh. The intestine wall was also examined to collect attached acanthocephalans. All parasites were collected, counted and preserved in 4% formaldehyde (*n* = 23), 70% ethanol (*n* = 27), or in 90% ethanol (*n* = 10).

The intestine of five South American fur seals *Arctocephalus australis* Zimmerman and 15 South American sea lions *Otaria flavescens* Shaw, found dead on northern Patagonian beaches (Fig 1B) between 1998 and 2009 (see [43] for details) were also surveyed for *C*. *australe* using the same procedure described above. Acanthocephalan specimens were washed in saline, counted, and preserved in 70% ethanol.

All acanthocephalan specimens were sexed. Female worms were classified into two categories [45]: juvenile (having ovarian balls only) and adult (with at least some fully formed eggs). A total of 20, 30 and 30 females were randomly selected from 12 penguins, 3 sea lions and 4 fur seals, respectively. Trunk length, disk diameter, and body volume (assuming a conical shape) of these specimens were measured following Hernández-Orts et al. [46]. Fecundity was estimated as the number of acanthors per female [47] in 10, 30 and 30 gravid females, randomly selected, from 4 penguins, 3 sea lions and 4 fur seals, respectively. The contents of each worm were washed into a beaker and the volume made up to 10 ml, then continually agitated using a magnetic stirrer for at least 5 min. Ten separate samples of 10 μl were taken and the number of developing and fully developed eggs counted. Fully developed eggs were distinguished by having a completely formed acanthor inside. Mean numbers of each egg type obtained from these samples were then extrapolated to the total volume [47].

### Morphological description

Ten acanthocephalans (two males and eight females) from Magellanic penguins fixed in 70% ethanol were punctured with a fine needle, stained with Mayer’s hematoxylin, washed in distilled water, dehydrated in ethanol, cleared in methyl salicylate and mounted in Canada Balsam. Specimens were examined using a compound microscope equipped with bright field and differential interference contrast optics. Morphometric measurements were taken using the Leica Application Suite microscope imaging software. Measurements are given in mm unless otherwise stated. Fifteen fully developed eggs were drawn *in situ* through the body wall of female worms and measured. Voucher specimens are deposited in the National Helminth Collection, Biology Institute, National Autonomous University of Mexico, Mexico City (CNHE) and the Helminthological collection of the Oswaldo Cruz Institute (CHIOC), Rio de Janeiro, Brazil.

Four specimens (two females and two males) were also examined with scanning electron microscopy (SEM). They were dehydrated through an ethanol series, critical point dried and coated with a gold-palladium alloy to a thickness of 250 nm. Specimens were examined with a Hitachi 4100 FE scanning electron microscope, operating a 20 kV, from the Central Service for the Support to Experimental Research (SCSIE) of the University of Valencia.

### DNA extraction, PCR amplification and sequencing

Total genomic DNA was isolated from single ethanol-fixed specimens following a Chelex® protocol as described in Georgieva et al. [48]. The targeted partial 28S rRNA (domains D1–D3) and partial fragments of the mitochondrial cytochrome *c* oxidase subunit 1 (*cox*1) genes were amplified using primer combinations of ZX-1 (forward; 5'-ACC CGC TGA ATT TAA GCA TAT-3'; [49]) and 1500R (reverse; 5'-GCT ATC CTG AGG GAA ACT TCG-3'; [50]) for the 28S rDNA and #507 (forward; 5'-AGT TCT AAT CAT AA(R) GAT AT(Y) GG-3'; [51]), and HC02198 (reverse; 5'-TAA ACT TCA GGG TGA CCA AAA AAT CA-3'; [52]) for *cox*1. Cycle reactions were carried out in 25 μl total reaction volumes using illustra puReTaq Ready-To-Go PCR beads (GE Healthcare, Buckingamshire, UK) and 2 μl of 5 pmol of each primer. Cycling conditions for the 28S rDNA amplifications were as follows: initial denaturation at 95°C for 5 min, followed by 40 cycles consisting of (95°C for 30 sec, annealing at 55°C for 30 sec, extension at 72°C for 2 min) and final extension hold at 72°C for 7 min. For *cox*1 amplifications, the cycling conditions included: initial denaturation at 94°C for 5 min, followed by 35 cycles of denaturation-annealing extension schedule (94°C for 1 min, annealing at 40°C for 1 min, and extension at 72°C for 1min) and a final extension step at 72°C for 5 min. PCR amplicons were purified directly using Qiagen QIAquickTM PCR purification Kit (Qiagen Ltd, UK) and sequenced for both strands using the same primers as used in the PCR reactions. Sequencing was performed on an ABI Prism 3130xl automated sequencer using ABI Big Dye chemistry (ABI Perkin-Elmer). Contiguous sequences were assembled and edited in MEGA v.7 [53] and representative sequences were deposited in GenBank under accession numbers MF497330–MF497335.

The newly-obtained sequences were aligned in two independent alignments together with published representative 28S rDNA and *cox*1 sequences for species of *Corynosoma* (Table 1) [54–58]. Alignments were constructed using MAFFT v. 7 [59] on the EMBL-EBI bioinformatics platform [60]. The trimmed alignments comprised 792 (28S rDNA dataset) and 573 (*cox*1 dataset) nucleotides (nt) positions. *Cox*1 dataset was aligned with reference to the amino acid translation, using the invertebrate mitochondrial code (transl_table = 5; [61]). Pairwise genetic distances (p-distance model, i.e. the percentage of pairwise character differences with pairwise deletion of gaps) were calculated with MEGA v.7.

**Table 1:** Taxa included in the phylogenetic analyses with data on host, locality and GenBank accession number.

Species	Host	Locality	GenBank accession number		Source
			28S rDNA	*cox*1	
**Genus *Corynosoma* Lühe, 1904**					
*C*. *australe* Johnston, 1937	*Arctocephalus australis* Zimmermann	Northern Patagonia, Chubut (Argentina)	MF497330	MF497333	Present study
	*Otaria flavescens* Shaw	Northern Patagonia, Chubut (Argentina)	–	KX957714	[39]
		Northern Patagonia, Chubut (Argentina)	MF497331	MF497334	Present study
	*Phocarctos hookeri* (Gray)	Enderby Island (New Zealand)	JX442180	JX442191 ^a^	[54]
	*Spheniscus magellanicus* (Forster)	Rio de Janeiro (Brazil)	MF497332	MF497335	Present study
*C*. *enhydri* Morozov, 1940	*Enhydra lutris* (L.)	Monterey Bay, California (USA)	AY829107	DQ089719	[55, 56]
*C*. *hannae* Zdzitowiecki, 1984	*Colistium guntheri* (Hutton)	Otago, South Island (New Zealand)	–	KX957724	[39]
	*Peltorhamphus novaezeelandiae* Günther	Otago, South Island (New Zealand)	–	KX957726	[39]
	*Leucocarbo chalconotus* (Gray)	Otago Harbour, South Island (New Zealand)	–	KX957718	[39]
	*Phalacrocorax punctatus* (Sparrman)	Otago Harbour, South Island (New Zealand)	–	KX957722	[39]
	*P*. *hookeri*	Enderby Island (New Zealand)	–	KX957715	[39]
*C*. *obtuscens* Lincicome, 1943	*Callorhinus ursinus* L.	St. Paul Island, Alaska (USA)	JX442181	JX442192	[54]
*C*. *magdaleni* Montreuil, 1958	*Phoca hispida saimensis* (Nordquist)	Lake Saimaa (Finland)	EU267815	EF467872	[57, 58]
*C*. *strumosum* (Rudolphi, 1802)	*Phoca hispida botnica* Gmelin	Baltic Sea (Finland)	–	EF467871	[58]
	*Phoca vitulina* L.	Monterey Bay, California, (USA)	EU267816	EF467870	[57, 58]
*C*. *validum* Van Cleave, 1953	*C*. *ursinus*	St. Paul Island, Alaska (USA)	JX442182	JX442193	[54]
Outgroup					
**Genus *Bolbosoma* Porta, 1908**					
*B*. *turbinella* (Diesing, 1851)	*Eschrichtius robustus* Lilljeborg	Monterey Bay, California (USA)	JX442178	JX442189	[54]

^a^Identified as *C*. *hannae* by Hernández-Orts et al. [39].

Maximum likelihood (ML) and Bayesian inference (BI) analyses were carried out in order to assess the species boundaries in phylogenies inferred separately from the nuclear and mitochondrial datasets. jModeltest 2.1.9. [62] was used to select the appropriate models of evolution prior to analyses under the Akaike Information Criterion with a correction for small sample sizes (AICc) [63,64]. This was the HKY+Г for both datasets. MrBayes 3.2.6 [65] was used for the BI analyses and run on the CIPRES Science Gateway v.3.3 (http://www.phylo.org/sub_sections/portal/) [66] on two parallel runs with random starting trees. Log likelihoods were estimated over 10,000,000 generations with every 1,000th tree sampled. The 'burn-in' period was set for the first 25% of the generated trees. Data convergence and stationarity distribution of the runs were assessed in TRACER v.1.5. [67]. Maximum likelihood analyses were run on PhyML v.3.0 [68] as an online execution on the ATGC bioinformatics platform (http://www.atgc-montpellier.fr/phyml/). The nodal support was estimated over 1,000 bootstrap pseudoreplicates. Resulted trees were visualised with FigTree v.1.4.2 [69].

### Comparative analyses

Infection parameters of *C*. *australe* were estimated following Bush et al. [70] and Rózsa et al. [71]. The 95% confidence interval (CI) for prevalence and overall sex ratio (percent males in the sample) was calculated with Sterne’s exact method [72], and for mean intensity, mean abundance, and mean sex ratio per host with the bias-corrected and accelerated bootstrap method using 20,000 replications [71,73].

Differences between host species in the prevalence of *C*. *australe*, as well as the sex ratio and proportion of gravid females in the overall sample (i.e. at component population level), were tested with Fisher's tests. Values of intensity, and sex ratio and percent gravid females per host (i.e. at infrapopulation level) were compared with Kruskal-Wallis tests with *post hoc* comparisons [74]. A mixed permutational MANOVA based on 20,000 permutations of the matrix of Euclidean distances was used to test for differences of trunk length and disk diameter of female *C*. *australe*; ‘host species’ and ‘host individual’ were treated as a fixed and random factors, respectively (see [75], for details). Likewise, a mixed PERMANCOVA was used to investigate differences in the number of developing and fully developed eggs between host species; ‘host individual’ was treated as a random factor, and ‘body volume’ as fixed covariate to control for the effect of body size on fecundity values.

Infection parameters were obtained with the statistical software Quantitative Parasitology v3.0 [71,73], the permutational MANOVA and MANCOVA were carried out with PERMANOVA for Primer [75], and the remaining analyses with statistical package SPSS v.23 for Windows. Statistical significance was set at *P* < 0.05.

## Results

### Morphological description of *C*. *australe* from *S*. *magellanicus*

***Corynosoma australe* Johnston, 1937.** Synonym *Corynosoma otariae* Morini and Boero, 1960.

**Fig 2:**
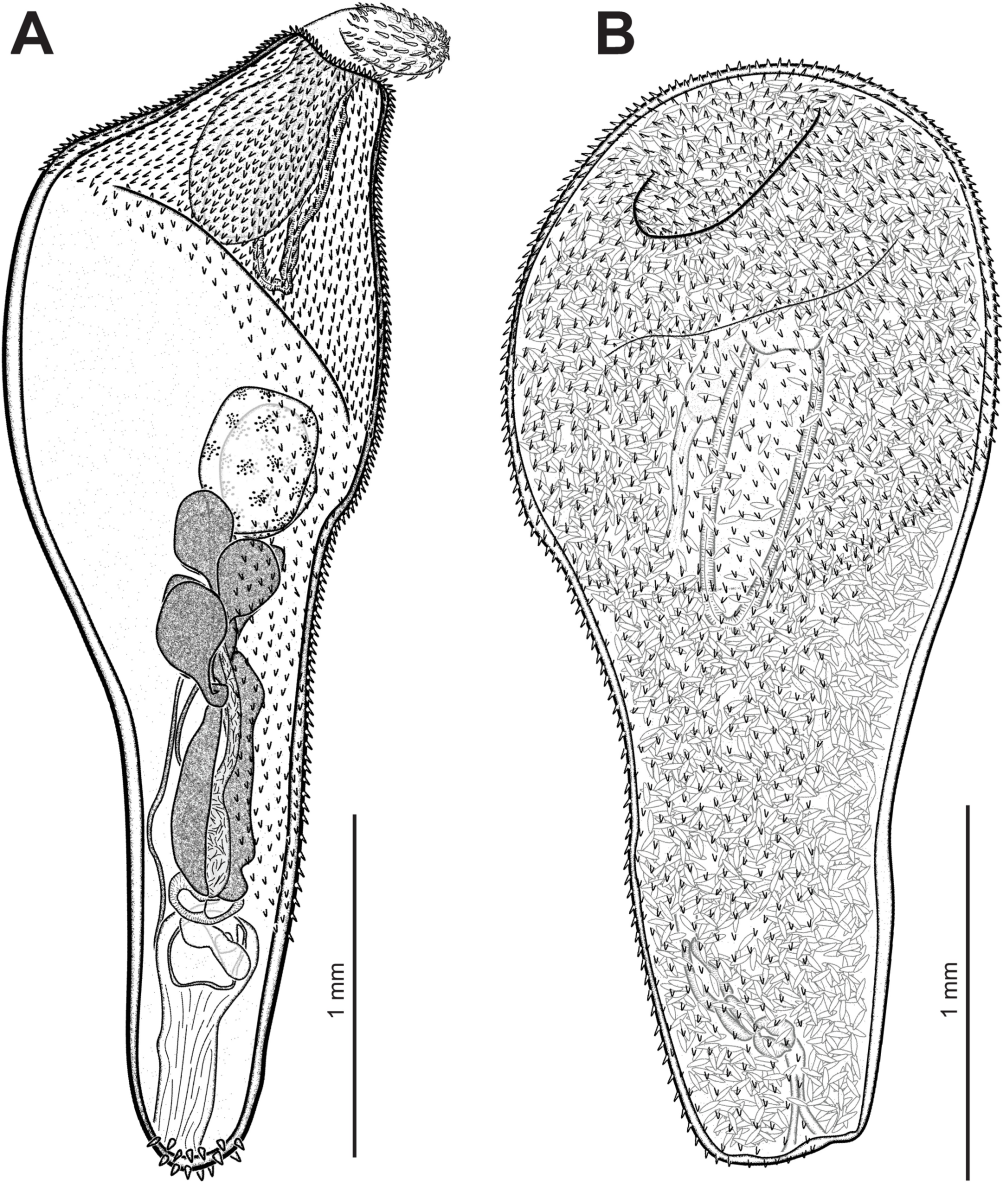
*Corynosoma australe* collected from *Spheniscus magellanicus* off the Brazilian coasts. **A** Adult male, whole worm, lateral view, voucher. **B** Adult female, whole worm, ventral view, voucher.

**Fig 3:**
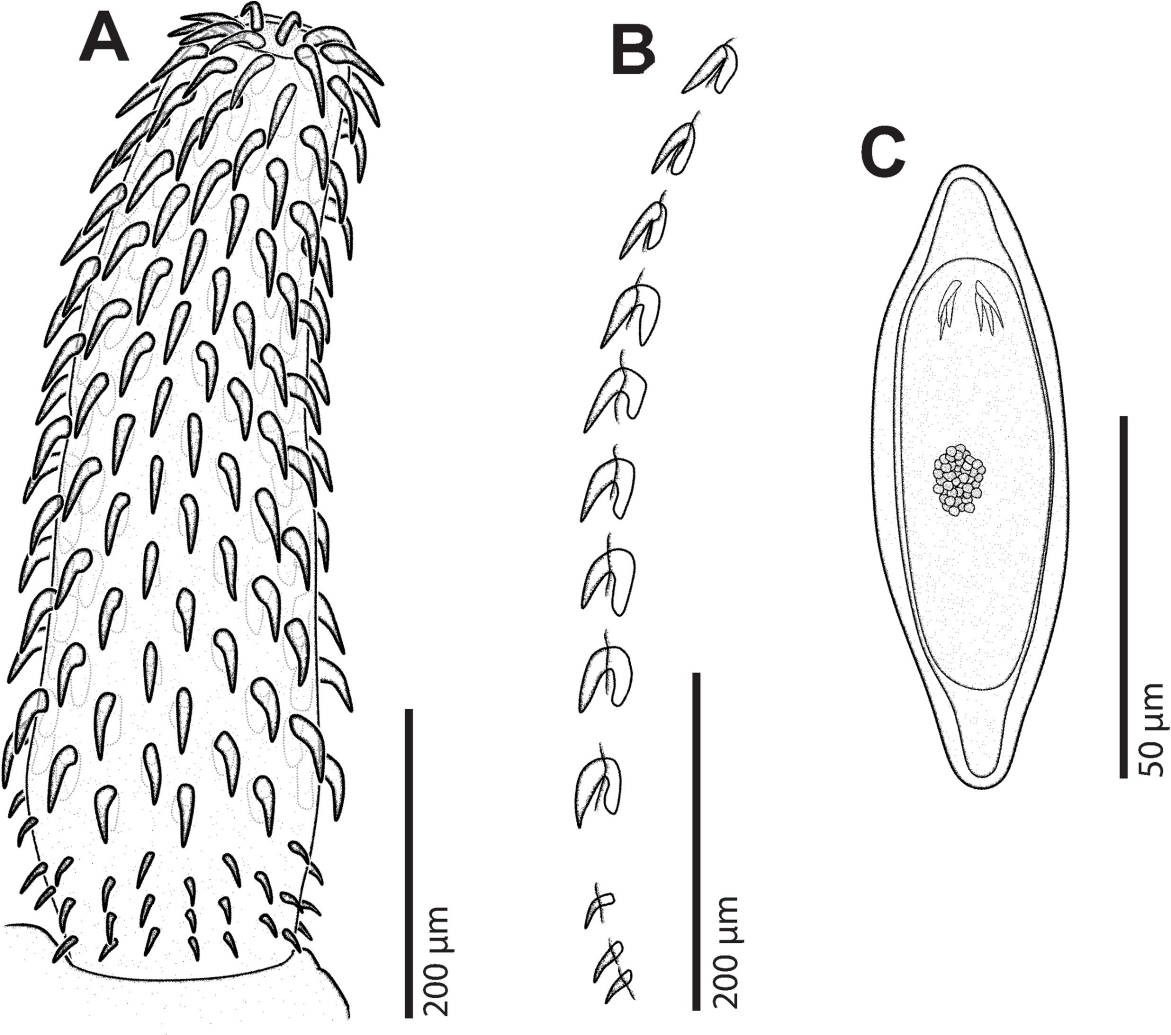
*Corynosoma australe* collected from *Spheniscus magellanicus*. **A** Proboscis of juvenile female, voucher. **B** Hooks of a longitudinal row of juvenile female, lateral view. **C** Egg.

**Fig 4:**
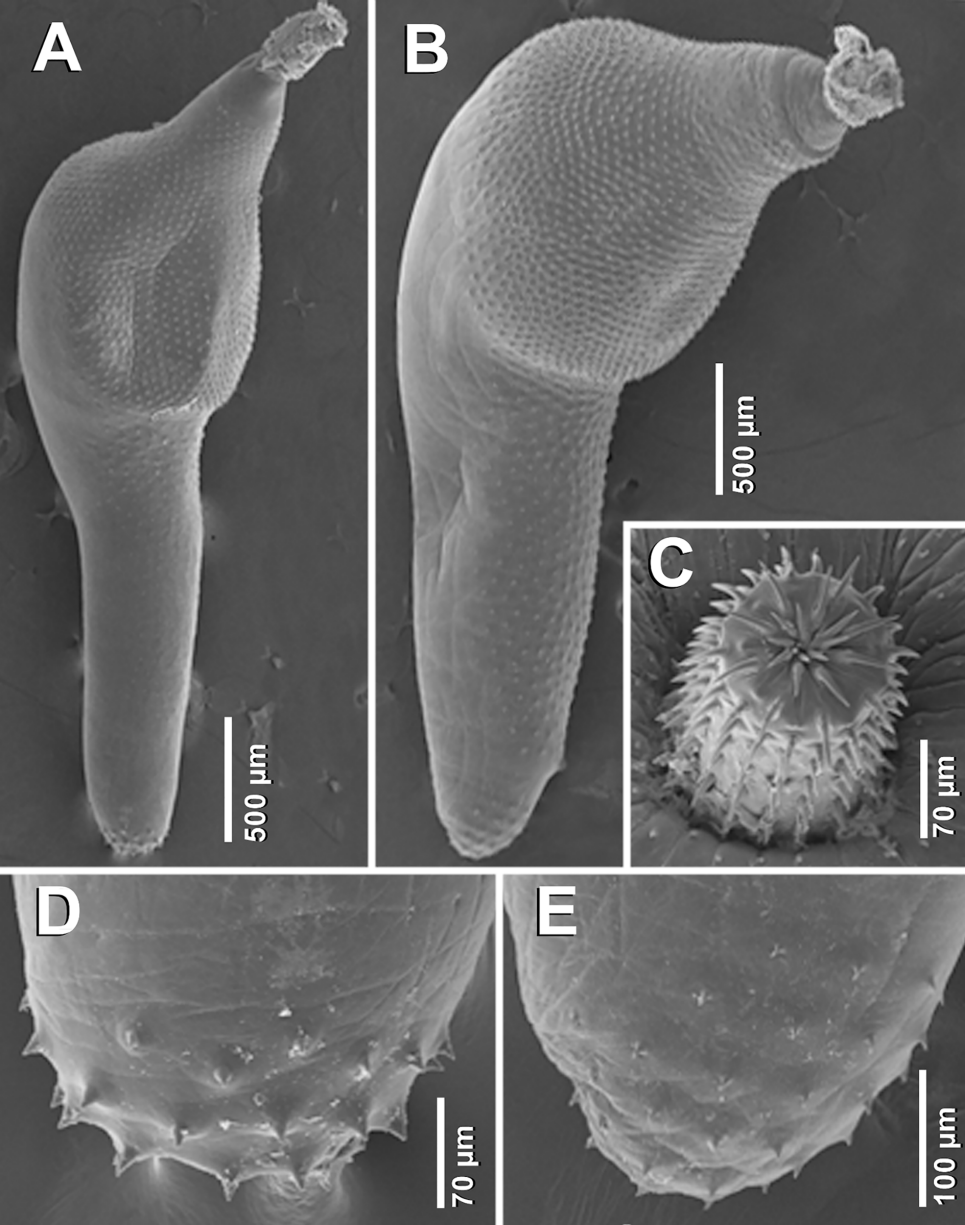
Scanning electron micrographs of *Corynosoma australe* collected from *Spheniscus magellanicus* off Brazil. **A** Adult male, whole worm, lateral view. **B** Adult female, whole worm, lateral view. **C** Female proboscis, subapical view, distal hooks invaginated. **D** Posterior end of adult male showing genital spines, lateral view. **E** Posterior end of adult female showing genital spines, lateral view.

*General* [Based on 2 mounted adult males, 3 gravid females and 5 juvenile females with ovarian balls; 2 adult males and 2 females for SEM]. Specimens white to yellowish. Females slightly larger than males (Fig 2A and 2B). Proboscis cylindrical. Hooks arranged in 19–20 rows, each row comprising 12–14 hooks, 9–11 anterior hooks and 3–4 small basal hooks (Figs 3A, 3B and 4C). Combinations of anterior/basal hooks (only in fully everted proboscis (*n* = 2), as follows: 9/3, 10/3 and 11/3. Neck trapezoid. Trunk expanded anteriorly into a disk. Hind-trunk elongated posteriorly. Fore-trunk shorter than hind-trunk [fore-trunk to hind-trunk length ratio 1:2.17–2.95 for males, 1:1.79–2.17 for females]. Trunk armed with spines, covering dorsally the anterior part of the disk, and spreading ventrally onto three quarters of the hind-trunk in males (Figs 2A and 4A), and almost reaching genital spines in females (Fig 4B). Genital spines present in both sexes (Figs 2A, 4D and 4E). Proboscis receptacle double-walled, with ellipsoidal cephalic ganglion at its posterior end. Lemnisci equal in size, shorter or similar in size than proboscis receptacle. Genital pore terminal in males and subterminal in females.

*Male* [Based on 2 mounted specimens and 2 for SEM]. Body 3.91–4.22 long. Trunk 3.06–3.41 long; fore-trunk 0.77–1.02 long; hind-trunk 2.21–2.39 long. Disk 1.17–1.29 long. Somatic armature covering 68–72% of trunk length; trunk spines 35–38 μm long. Neck 0.15–0.26 × 0.32–0.34. Proboscis 0.58–0.63 × 0.20–0.21; proboscis receptacle 0.66–0.67 × 0.17–0.18. Lemnisci 0.61–0.68 long. Testes ovoid, parallel, 0.36–0.51 × 0.27–0.34. Cement glands claviform, 6 in 3 pairs, 0.19–0.28 × 0.16–0.26. Säfftigen’s pouch 0.60–0.64 long. Genital spines 41–45µm long.

*Female* [Based on 3 gravid females and 2 females for SEM]. Body 4.23–4.80 long. Trunk 3.36–3.90; fore-trunk 1.06–1.34 long; hind-trunk 2.30–2.64 long. Disk 1.35–1.60 long. Somatic armature covering 82–89% of trunk length; trunk spines 32–38 μm long. Neck 0.15–0.20 × 0.34–0.40. Proboscis 0.70–0.72×0.19–0.20; proboscis receptacle 0.80–1.03 × 0.19–0.21. Lemnisci 0.70–0.96 long. Genital spines 32–36 μm long. Mature eggs, containing a fully developed acanthor, 83–89 × 27–33 μm (Fig 3C).

*Definitive host*: Magellanic penguin *Spheniscus magellanicus* (Forster) (Aves: Spheniscidae) (new host record).

*New localities*: Arraial do Cabo, Rio de Janeiro and Rio Grande do Sul, Brazil.

*Site in host*: Intestine.

*Voucher material*: CNHE 10448; CHIOC 38874–38877.

*Representative DNA sequences*: ex *S*. *magellanicus* MF497332 (28S rDNA), MF497335 (*cox*1); ex *O*. *flavescens* MF497331 (28S rDNA), MF497334 (*cox*1); ex *A*. *australis* MF497330 (28S rDNA), MF497333 (*cox*1).

Other records.

*Type-hosts*: *Neophoca cinerea* (Péron). Johnston and Mawson [76] clarified that the sea lion identified as *A*. *forsteri* in Johnston [77] was actually *N*. *cinerea*].

*Other definitive hosts*: *Hydrurga leptonyx* (de Blainville), *Phocarctos hookeri* (Gray), *Otaria flavescens* (Shaw), *Arctocephalus forsteri* (Lesson), *A*. *tropicalis* (Gray), *A*. *pusillus* (Schreber), *A*. *australis* (Zimmermann) and *Mirounga leonina* (L.) (see S1 dataset).

*Remarks*: The specimens collected from *S*. *magellanicus* share the diagnostic morphological traits of the genus *Corynosoma* concerning body shape, trunk spination, shape of lemnisci, and number and shape of cement glands [18,27]. These acanthocephalans were morphologically identified as *C*. *australe* based on the following distinguishing features [34,41]: (i) cylindrical proboscis; (ii) proboscis armed with 19–20 hook rows; (iii) rows with 9–11 anterior hooks and 3–4 small basal hooks; (iv) trunk size, (v) distribution of somatic spines; and (vi) genital spines well separated from the somatic spines. Molecular data generated in the present study supports that these acanthocephalans belongs to *C*. *australe* (see below).

Comparative morphometric data from all available descriptions of *C*. *australe* are given in Table 2. Specimens of *C*. *australe* from *S*. *magellanicus* appear to be most closely related to *C*. *australe* from otariids from the south-western Atlantic; they all share a higher upper limit for the number of hook rows (20 *vs* 18 in specimens from the south-eastern Pacific and Antarctica). Other metrical data of *C*. *australe* from *S*. *magellanicus* fall within the ranges provided in previous descriptions (see Table 2).

**Table 2:** Metrical and meristic data of males and females of *Corynosoma australe* from different definitive hosts. Body length and trunk length in millimetres, other measurements in micrometres.

Reference	Johnston [77]	Johnston and Edmonds [78]	Morini and Boero [79]	Zdzitowieki [37]	Smales [34]	Smales [34]	Sardella et al. [41]	Present study
Hosts ^a^	1	2	3	4	5	6	7	8
Locality ^b^	1	2	3	4	5	6	7	8
**General**								
No. of rows of hooks	18	18	20	16–18	18	18	18–20	19–20
No. hook per row	13–14	12–14	12–14	11–15	13–14	13–14^c^	12–14	12–14
No. large hooks per row	1–10	11–12	9–11^c^	9–11	10–11	10–11^c^	9–11	9–11
No. small hooks per row	2–3	1–2	3	2–4	3–4	3–4^c^	2–4	3–4
**Female**								
Total length	3–4	–	4.5	3.4–4.5	–	–	4.2–5.5	4.23–4.80
Trunk length	–	2.5–3.1	3.7^c^	2.7–3.1	1.8–2.4	2.8–4.5	3.3–4.7	3.36–3.90
Proboscis length	700	580–610	700	549–731	–	560–760	600–740	700–720
Proboscis receptacle	1,100	600–900	–	1,020–1,290	850–1,120	1,050–1,350	840–1,004	800–1,030
Egg size (length × width)	75–85 × 23–29	72–86 × 19–23	90 × 28–30	66–82 × 23–32	88–96 × 32	84–96 × 28–36	92–115 × 27–42	83–89 × 27–33
**Male**								
Body length	3–4	3.45^b^	4.8	2.9–3.7	–	–	4.2–5.4	3.91–4.22
Trunk length	5.7^c^	2.6–2.9	3.8^c^	2.2–2.9	2.1–3.0	2.4–3.5	3.4–4.3	3.06–3.41
Proboscis length	700	540–600	400	545–654	560	540–760	580–720	580–630
Proboscis receptacle	1,100	600–900	1400	900–1050	650–1,100	1,100–1,250	740–920	660–670
Testes size (length × width)	400 (diam.)	200–400 (diam.)	–	360–550 × 290–420	240–400 × 270–408	288–400 × 336–400	400–700 × 260–420	367–514 × 270–349

^a^ 1. *Neophoca cinerea* (Péron) (Johnston and Mawson [76] clarified that the sea lion identified as *A*. *forsteri* in Johnston [77] was actually *N*. *cinerea*); 2. *Phocarctos hookeri* (Gray); *Hydrurga leptonyx* (de Blainville); 3. *Otaria flavescens* (Shaw); 4. *Hydrurga leptonyx*; 5. *Neophoca cinerea*; 6. *Arctocephalus pusillus* (Schreber); 7. *Arctocephalus australis* (Zimmermann); 8. *Spheniscus magellanicus* (Forster)

^b ^1. Pearson Island (Australia); 2. Campbell Island; Auckland Islands (New Zealand); 3. Argentina; 4. King George Island, South Shetlands (Antarctic); 5. Pearson Island, Greenly Island, Port Adelaide, Dangerous Reef (Australia); 6. Phillip Island (Australia); 7. Claromecó; San Clemente del Tuyú (Argentina); 8. Arraial do Cabo, Rio de Janeiro; Rio Grande do Sul (Brazil)

^c ^Estimated from the published drawing.

To date, four nominal species of *Corynosoma* have been reported from the intestine of penguins: *C*. *bullosum* (v. Linstow, 1892) from *Pygoscelis papua* (Forster); *C*. *hamanni* Linstow, 1892 from *P*. *papua* and *Pygoscelis adeliae* (Hombron & Jacquinot); *C*. *pseudohamanni* from *Pygoscelis antarcticus* (Forster) and *P*. *adeliae*; and *C*. *shackletoni* Zdzitowiecki, 1978 from *P*. *papua* (see [80]). An unidentified species of *Corynosoma* was reported from *S*. *magellanicus* by Boero et al. [81] and Diaz et al. [82] off the coast of Argentina. Specimens of *C*. *australe* from *S*. *magellanicus* from Brazil are somewhat smaller in length (3.9–4.8 *vs* 4.5–5.0 mm) than *Corynosoma* sp. described by Boero et al. [81], but all specimens have similar number of hooks per row (9–11 anterior hooks and 3–4 small basal hooks). The specimens of *Corynosoma* sp. reported by Diaz et al. [82] from *S*. *magellanicus* in Patagonia clearly differ from our material by having: (i) a proboscis nearly cylindrical with a swollen base, and 16 longitudinal rows with 6–7 anterior hooks per row, and (ii) a non-spined area on the disk (see figure 10 in Diaz et al. [82]). These specimens actually resemble those of *Andracantha* sp. reported by Hernández-Orts et al. [43] in sympatric pinnipeds.

Distribution (following Spalding et al. [83]): Southwest Australian Shelf, sub-Antarctic New Zealand, warm temperate south-western Atlantic, temperate southern Africa and Scotia Sea, Antarctica (see S1 dataset).

### Molecular analyses

**Fig 5:**
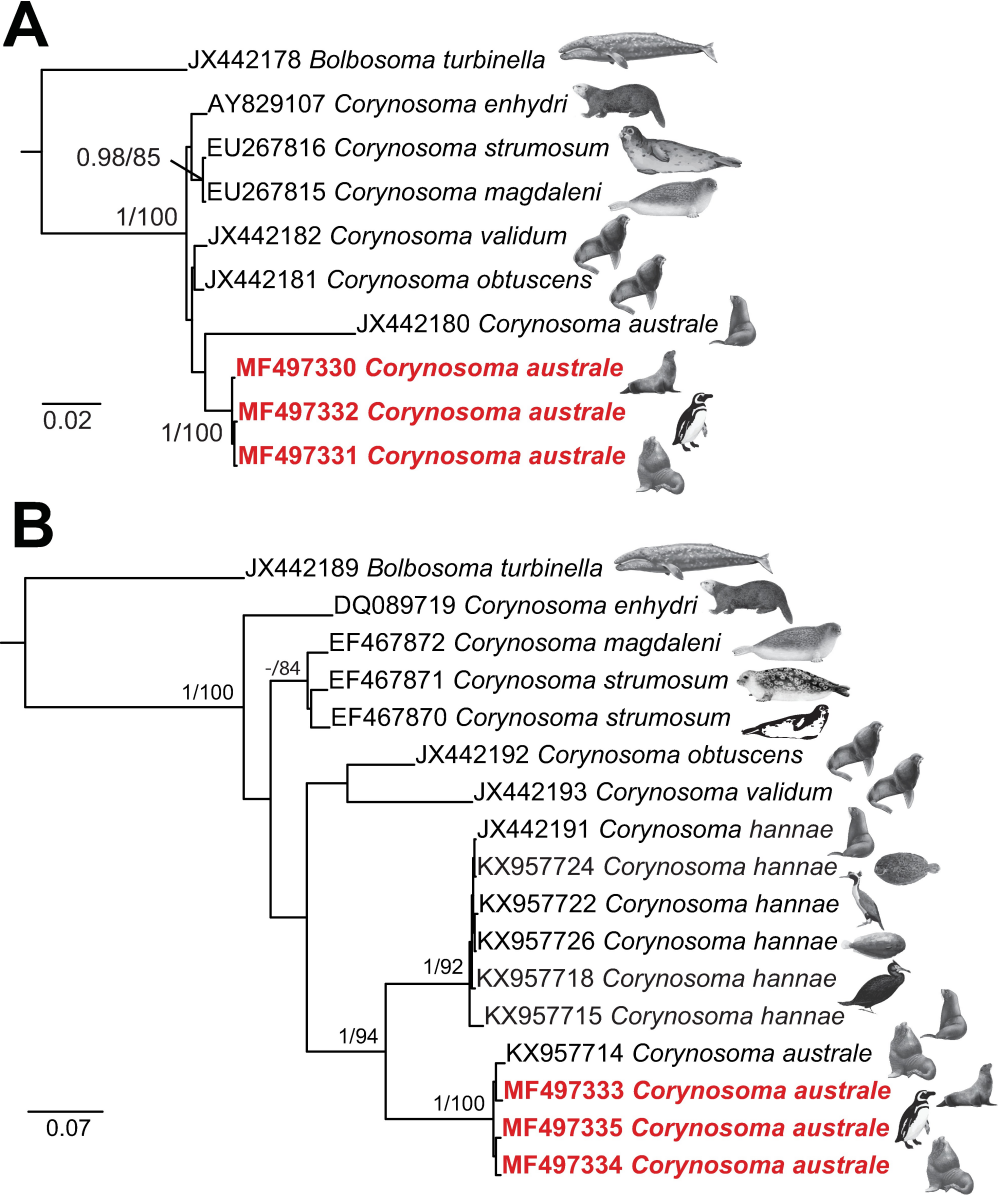
Bayesian inference (BI) phylograms for *Corynosoma* inferred from (**A)** 28S rRNA and (**B)** mitochondrial *cox*1 datasets. Nodal support is given as posterior probabilities (BI) and bootstrap values from Maximum likelihood (ML) analyses as (BI/ML); only support values > 0.95 (BI) and > 75% (ML) are shown. The scale-bar indicates the expected substitutions number per site. The hosts from which the isolates were collected are shown on the trees (see Table 2 for details). The newly generated sequences are presented in red. Outgroup: *Bolbosoma turbinella*.

Our study generated 3 identical partial sequences for the 28S rDNA (1,083–1,088 nt in length) ribosomal gene and 3 partial sequences for the *cox*1 (573 nt in length; intraspecific sequence divergence range 0.7–1.0%; 4–6 nt difference) mitochondrial gene for specimens of *C*. *australe* collected from *A*. *australis* and *O*. *flavescens*, both sampled from Patagonia, Argentina, and specimens collected from *S*. *magellanicus* recovered at the coast of the State of Rio de Janeiro in Brazil. Sequence divergence for both datasets was estimated for all representatives of the genus. Maximum likelihood and Bayesian inference analyses yielded trees with similar topologies for both, the nuclear and mitochondrial gene datasets. The resultant bootstrap values from the ML analyses are summarised and presented together with the posterior probabilities on the Bayesian phylograms. The phylogenies inferred from the 28S rDNA dataset (Fig 5A) recovered the novel isolates for *C*. *australe* in a strongly-supported clade. The isolate for *C*. *australe* (JX442180) of García-Varela et al. [54] branched out from the later clade, albeit with lack of support. This result further confirms the distinct species status of the isolate of García-Varela et al. [54] from *P*. *hookeri*, as recently indicated by Hernández-Orts et al. [39], and assigned to *C*. *hannae* based on the *cox*1 sequence data. The relationships of the remaining species, i.e. *C*. *validum*, *C*. *obtuscens*, *C*. *enhydri*, *C*. *magdaleni* and *C*. *strumosum* were poorly resolved. The latter two species formed the only well-supported grouping with high nodal support.

*Corynosoma* species relationships yielded from the *cox*1 dataset (Fig 5B) were highly concordant with those of Hernández-Orts et al. [39], although with poor support for most of the nodes. The newly sequenced isolates clustered together with an isolate for *C*. *australe* of Hernández-Orts et al. [39] from *O*. *flavescens* off Patagonia in a strongly supported clade sister to the *C*. *hannae* clade. The remaining relationships were poorly resolved with the only well-supported clade formed by the two isolates for *C*. *strumosum* from the ringed and harbour seals and an isolate for *C*. *magdaleni* collected from the Saima ringed seal. Isolates for *C*. *obtuscens* and *C*. *validum* clustered in a clade with lack of support and the isolate for *C*. *enhydri* from the sea otter branched out as the earliest diverging taxon to the representatives of *Corynosoma* spp., although with moderate support.

The overall intraspecific divergence for the species of *Corynosoma* ranged between 0.6–6.5% (6–51 nt difference) in the 28S dataset and between 3.1–17.3% (18–98 nt difference) in the *cox*1 dataset. The isolates for *C*. *hannae* had the lowest sequence divergence compared to *C*. *australe* for both datasets, i.e. 6.3% (49 nt difference) in the 28S and 12.9–14.3% (74–82 nt difference) in *cox*1, respectively. The recently published single *cox*1 sequence for an isolate of *C*. *australe* from *O*. *flavescens* [39] differed by 1.2% (7 nt difference) from the three novel isolates studied.

### Comparative analyses

The prevalence of *C*. *australe* in *S*. *magellanicus* varied significantly among years. None of the 44 penguins examined in 2008 (32 from Rio de Janeiro and 12 from Sergipe) was infected. However, 17 out of 39 penguins collected in Rio de Janeiro in 2010 (prevalence: 39.5%) and 1 out of 4 penguins collected in Rio Grande do Sul in 2011 (prevalence: 25.4%) harboured *C*. *australe*. The difference of prevalence in Rio de Janeiro between 2008 and 2010 was highly significant (Fisher’s test, *P* < 0.0001).

The overall prevalence of *C*. *australe* was significantly lower in *S*. *magellanicus* (39.5%) than in *O*. *flavescens* (100%) and *A*. *australis* (80%) (Fisher’s tests, *P* < 0.01 in both comparisons), but the two latter did not differ from one another (*P* = 0.25). The intensity of infection also differed significantly among host species (Kruskal-Wallis test, *χ*^2^ = 24.7, 2 *df*, *P* < 0.0001). The *post hoc* test indicated only highly significant differences between penguins and pinnipeds (*P* < 0.001 in both tests). The intensity of *C*. *australe* was two to three orders of magnitude lower in penguins than in pinnipeds (Table 3).

**Table 3:** Populational data of *Corynosoma australe* in three host species collected in South American waters, *Spheniscus magellanicus*, *Otaria flavescens* and *Arctocephalus australis*.

Parameter	n	*S*. *magellanicus* (N = 17)	n	*O*. *flavescens* (N = 15)	n	*A*. *australis* (N = 4)
Mean intensity	60	3.5 (2.1–7.4)	22,347	1481.7 (796.8–2598.1)	1,392	348.0 (161.3–521.5)
Overall sex ratio (%) (95% CI)	60	6.7 (2.3–16.4)	22,347	49.8 (47.8–51.9)	1,392	42.8 (40.2–45.4)
Mean sex ratio (95% CI)	60	9.6 (2.0–30.0)	22,347	49.7 (44.4–55.0)	1,392	40.6 (28.9–45.4)
Overall % gravid females (95% CI)	59	32.1 (21.2–45.5)	11,208	96.1 (95.0–97.2)	796	94.0 (92.1–95.5)
Mean % gravid females (95% CI)	59	15.3 (6.2–28.4)	11,208	88.8 (68.0–96.0)	796	90.5 (76.2–96.1)
Mean trunk length (mm) ± SD	20	2.79 ± 0.28	30	2.30 ± 0.37	30	2.66 ± 0.26
Mean disk diameter (mm) ± SD	20	1.64 ± 0.18	30	1.50 ± 0.26	30	1.70 ± 0.18
Mean body volume (mm^3^) ± SD	20	2.01 ± 0.55	30	1.46 ± 0.24	30	2.06 ± 0.19
Mean fecundity ± SD	10	18,626 ± 21,838	30	21,876 ± 15,376	30	45,380 ± 18,650
Percent developed eggs ± SD	10	24.6 ± 23.4	30	35.5 ± 8.5	30	41.2 ± 9.6

*Abbreviations*: *n*, the sample of *C*. *australe* used in each calculation; N, host sample size.

At both the component population level (Fisher’s test, *P* < 0.0001) and infrapopulation level (Kruskal-Wallis H-test: *χ*^2^ = 21.7, 2 *df*, *P* < 0.0001) the sex ratio of *C*. *australe* differed significantly among host species; the *post hoc* test revealed only significant differences between penguins and the two pinniped species (*P* < 0,006 in all tests). The sex ratio was strongly biased against males in *S*. *magellanicus*, but it was close to 1:1 in both *O*. *flavescens* and *A*. *australis* (Table 3). A similar pattern of differences was found in the proportion of gravid females (Table 3). A substantial proportion of females of *C*. *australe* from S. *magellanicus* was gravid (> 30%, found in 6 penguins). However, this proportion was significantly greater in pinnipeds, i.e. > 90% (Kruskal-Wallis H-test: *χ*^2^ = 23.7, 2 *df*, *P* < 0.0001; *post hoc* comparisons: *S*. *magellanicus vs A*. *australis* [*P* < 0.001]; *S*. *magellanicus vs O*. *flavescens* [*P* < 0.001]; *A*. *australis vs O*. *flavescens* [*P* = 0.530]) (Table 3).

Morphometric data of females of *C*. *australe* are shown in Table 3. The mixed PERMANOVA revealed highly significant differences of females among host species (*F*_(2,65)_ = 12.37, *P* = 0.00005). The pair-wise *post hoc* comparisons indicated that females collected from *O*. *flavescens* were significantly smaller than those from *S*. *magellanicus* (*t* = 3.71, 48 *df*, *P* = 0.0003) and *A*. *australis* (*t* = 4.34, 58 *df*, *P* = 0.00005); the two latter did not differ one from another (*t* = 1.38, 48 *df*, *P* = 0.161) (Table 3).

**Fig 6:**
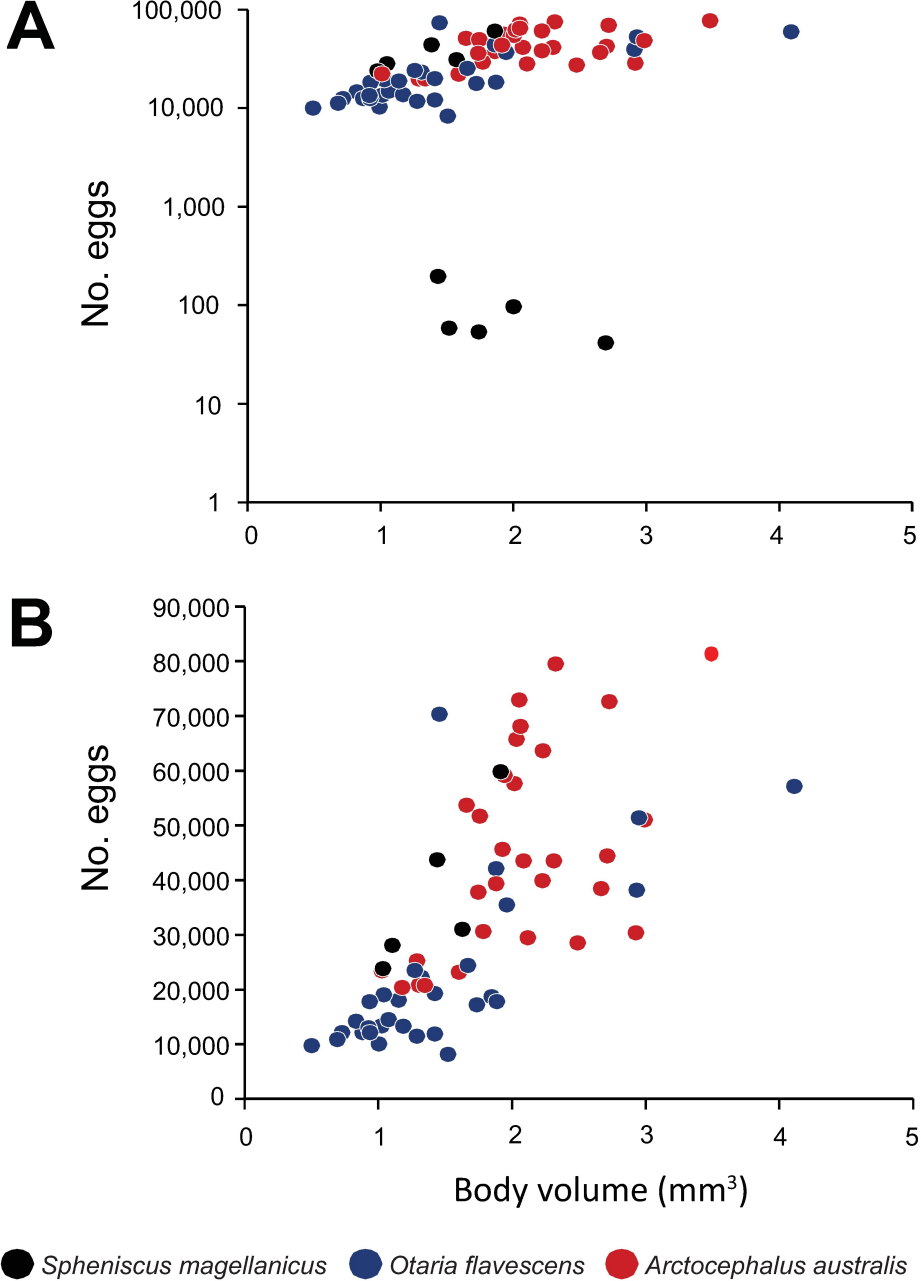
Distribution of fecundity values (number of eggs) and the body size (volume) *Corynosoma australe* from *Spheniscus magellanicus*, *Otaria flavescens* and *Arctocephalus australis*.

In the sample of 10 gravid females of *C*. *australe* from penguins used for fecundity analysis, two subsets of females were found: 5 of them harboured very small numbers of eggs (range: 41–195), compared to the other 5 (range: 23,700–59,700) (Fig 6A). The minimum fecundity observed in the samples of *C*. *australe* from *A*. *australis* and *O*. *flavescens* were 20,400 and 8,000 eggs, respectively. On average, fecundity was lower and more variable in females collected from *S*. *magellanicus* (Table 3). The difference was significant in the comparison with *A*. *australis* (Mann-Whitney test, *U* = 60, *n* = 40, *P* = 0.004), but not with *O*. *flavescens* (*U* = 127, *n* = 40, *P* = 0.488). Also, the proportion of fully developed eggs was the smallest in worms from *S*. *magellanicus* (Table 3). The PERMANCOVA was carried out excluding the 5 females from penguins with small number of eggs. There was a highly significant effect of body volume on fecundity (*F*_(1,56)_ = 44.85, *P* = 0.00005), and the slope of the regression did not differ among species (interaction ‘body volume’ * ‘species’: *F*_(2,56)_ = 1.39, *P* = 0.240) (Fig 6B). After controlling for body size, a significant ‘host species’ effect was detected (F_(2,56)_ = 4.73, *P* = 0.00061). The pair-wise comparisons indicated that females of *C*. *australe* from both *S*. *magellanicus* (*t* = 2.47, 22 *df*, *P* = 0.016) and *A*. *australis* (*t* = 2.76, 58 *df*, *P* = 0.0034) had significantly more eggs than those from *O*. *flavescens*, but they did not differ from one another (*t* = 0.53, 22 *df*, *P* = 0.749) (Fig 6B).

## Discussion

In addition to the morphological characterization of the specimens studied, molecular data confirm the conspecificity of the specimens of *C*. *australe* collected from the Magellanic penguin *S*. *magellanicus*, the South American fur seal *A*. *australis* and the South American sea lion *O*. *flavescens*. The phylogenies from both, 28S and *cox*1 datasets, suggest that *C*. *australe* has a wider host range infecting pinnipeds and sphenisciform birds in the Southern Hemisphere. Most significantly, our results call for further studies on the real host range and geographical distribution of *C*. *australe*. Our results from the 28S rDNA phylogenies confirmed the distinct species status of the isolate from the New Zealand sea lion reported as *C*. *australe* by García-Varela et al. [54] which most probably belongs to *C*. *hannae* as recently revealed by Hernández-Orts et al. [39] for the respective *cox*1 isolate.

*Corynosoma australe* has hitherto been reported in 16 marine mammals and birds, including 8 pinnipeds (6 otariids and 2 phocids), 7 cetaceans, and 1 shearwater [28]. Previous records indicate that the parasite was able to reproduce only in pinnipeds [28], with otariids exhibiting the highest prevalence and intensity of infection [28,43]. However, results from the present study indicate that Magellanic penguins also harbour a small reproductive population of *C*. *australe* in the south-western Atlantic, which represents an interesting finding because the parasite is unable to reproduce in potential sympatric definitive hosts that are phylogenetically much closer to pinnipeds, namely, cetaceans [28]. In what follows, we discuss this finding in an ecological and evolutionary context.

In the south-western Atlantic, populations of *C*. *australe* are sustained by two otariid species, i.e. *A*. *australis* and *O*. *flavescens* (e.g. [43,45,84]). The first intermediate host of *C*. *australe* is unknown but, in this region, this acanthocephalan occurs in a number of fish species, particularly from the benthic-demersal realm [25,45,85]. The broad use of the food web guarantees frequent transmission to *O*. *flavescens* and *A*. *australis*, both of which are opportunistic, broad-spectrum feeders (e.g. [86,87]). However, larvae of *C*. *australe* can also contact a wide array of sympatric piscivorous predators, including elasmobranchs [88], cetaceans [28] or Magellanic penguins (this study).

The infection levels of *C*. *australe* in penguins were far lower than those from typical otariid hosts. The extent to which such low infection levels result from the influence of contact *vs* compatibility filters (*sensu* [7]) is very difficult to elucidate, but the role of the former should not be underestimated. Firstly, in the same locality (Rio de Janeiro) there were sharp differences of prevalence of *C*. *australe* in penguins between years, suggesting that consumption of infected prey varied from year to year. Secondly, Magellanic penguins feed mainly on preys which are known to harbour very small numbers of *C*. *australe*. For instance, 6 prey taxa dominate the diet along the breeding areas of northern and central Patagonia in Argentina (e.g. [89–91]). Three of them are pelagic fish, i.e. Argentine anchovy *Engraulis anchoita* Hubbs and Marini, Argentine hake *Merluccius hubbsi* Marini, and silverside *Odontesthes smitti* (Lahille), in which infection levels of *C*. *australe* are low or exceptional (e.g. [25,92,93], and references therein). Magellanic penguins also feed on two pelagic and one demersal species of cephalopod, i.e. *Ilex* sp., *Loligo* sp., and *Octopus* sp., in which cystacanths of *Corynosoma* spp. have never been reported [94].

Further evidence on recruitment patterns comes from data on sex ratio. At the larval (cystacanth) stage, the sex ratio of *C*. *australe* is close to 1:1 [25], similarly as in most acanthocephalans, including other species of *Corynosoma* (e.g. [95–97]). However, in adult populations of acanthocephalans, females live longer than males and, therefore, populations tend to be female-biased [46, 96]. In fact, departures from a 1:1 sex ratio have been used as an index of the “age” of infections, with strongly female-biased samples indicating older infections [97]. The sex ratio of *C*. *australe* reported in otariids from the south-western Atlantic ranges from 30.8 [45] to 49.6 (this study). These differences likely reflect, at least to some extent, the rate of recruitment, with values closer to 1:1 indicating populations relatively more enriched with new recruits. Accordingly, the extremely female-biased sex ratio (6.0%) observed in penguins might reflect intense differential mortality of male *C*. *australe* in a sub-optimal host or, perhaps more likely, a very low rate of recruitment. It should be noted that the bird sample collected from Brazil was composed of wintering individuals that came from nesting colonies in Patagonia [98] (Fig 1A and 1B). Most of these penguins were anemic and starved during the peak of penguin mortalities in Brazil [99], thus suggesting that penguins might have acquired infections of *C*. *australe* long before they were collected; perhaps prior, or during, their winter migration.

We also found differences in the proportion of non-gravid females in *C*. *australe* populations, being significantly larger in Magellanic penguins than in otariids. Given the pattern of sex ratio values discussed above, this relative enrichment of non-gravid females in penguins can hardly be accounted for by a more recent recruitment of worms in these hosts. Rather, penguins seem to function as suboptimal hosts in which sexual maturation, and/or egg production of *C*. *australe*, are comparatively hampered. Regardless of maturity state, females of *C*. *australe* from penguins exhibited comparable, or even larger, body sizes than females collected from otariids. In contrast, the body size of worms collected from typical unsuitable hosts, namely, cetaceans, is nearly identical to that found in cystacanths [28]. This strongly suggests that, in penguins, females of *C*. *australe* are able to grow but, for unknown reasons, do not become gravid as readily as in otariids. In agreement with this, the proportion of developed eggs was also lower in females from penguins. However, the distribution of fecundity values exhibited a distinct pattern: some females harboured very few eggs, whilst other had very high fecundities (comparable, or even higher, that those estimated for worms collected from otariids). At present, the reasons for this striking pattern are difficult to elucidate; females with low and high fecundity were found in the same individual hosts, thus ruling out a pure effect of host individual [100,101].

### *Corynosoma australe* in Magellanic penguins as a case of host switch

As noted in the Background, successful host-switching events are more likely between ‘donor’ and ‘target’ hosts that are phylogenetically related [12]. This is clearly supported by patterns of specificity in species of *Corynosoma* (see S1 Table): (i) there is a single report of a species of *Corynosoma* from birds reproducing in mammals and none from mammals reproducing in birds; (ii) most reports of adult individuals of *Corynosoma* species from pinnipeds in other mammalian hosts involve carnivores; (iii) assuming that pinnipeds are original hosts for *Corynosoma* (e.g. [18,54]), host-switch with concomitant speciation was most frequent with other mammals than with birds.

Thus, how could the occurrence of adult *C*. *australe* in Magellanic penguins be interpreted? Since stochastic factors may play a significant role in the establishment of new associations, one possibility is that favourable mutations have allowed some individuals of *C*. *australe* to break the ‘compatibility’ filter, being able to colonize and to some extent persist in these phylogenetically unrelated avian hosts. Similar scenarios have been proposed, e.g., for *Contracaecum* nematodes in austral pinnipeds, which were apparently acquired from marine birds [102] or, at a higher taxonomic scale, species of brachycladiid digeneans, which are specific to cetaceans but originated from ancestors infecting fish [101]. However, it seems clear that species of *Corynosoma* colonized marine mammals from ancestors infecting aquatic birds [54]. Specifically, species of *Corynosoma* and *Bolbosoma* make up the sister clade of species of *Andracantha*, which are parasites of cormorants (and secondarily other marine birds) worldwide [54, 103]. Thus, there is the possibility that latent genetic variation resulting from a shared evolutionary history may generate a ‘sloppy fitness space’ [15] in *C*. *australe* that allows it to ecologically fit ancestral hosts. If so, the occurrence of *C*. *australe* in Magellanic penguins should be viewed as an example of phylogenetic conservatism *via* the use of a plesiomorphic (host) resource [14]. Assuming this hypothesis, the question why other marine birds (including cormorants) have never been reported to harbour adult individuals of other species of *Corynosoma* is intriguing, and likely illustrates the idiosyncratic nature of history.

## Supporting information

S1 Table Species of *Corynosoma* reported as adults in its typical hosts. Reports of adults in other host species are also included. Based on Aznar et al. [1], Dunagan and Miller [2], Amin [3], and references therein.
(DOC)

S1 dataset List of definitive hosts and localities of *Corynosoma australe* Johnston, 1937. (DOC)
