# Supplementary material for: From mammals back to birds: Host-switch of the acanthocephalan Corynosoma australe from pinnipeds to the Magellanic penguin Spheniscus magellanicus
Source: PLoS One. 2017 Oct 5;12(10):e0183809. doi: 10.1371/journal.pone.0183809 (PMC5628790; doi:10.1371/journal.pone.0183809)
Supplement: S1 Table — Reports of adults in other host species are also included. Based on Aznar et al. [1], Dunagan and Miller [2], Amin [3], and references therein. (DOC) [file pone.0183809.s001.doc]

**S1 Table.** Species of *Corynosoma* reported as adults in its typical hosts. Reports of adults in other host species are also included. Based on Aznar et al. [1], Dunagan and Miller [2], Amin [3], and references therein.

| **Species** | **Typical hosts** | **Other hosts** |
| --- | --- | --- |
| *C. australe*, *C. arctocephali*, *C. bullosum*, *C. caspicum, C. erignathi*, *C. evae*, *C. falcatum*, *C. gibsoni*, *C. hamanni*, *C. hannae*, *C. magdaleni, C. obtuscens, C. pseudohamanni*, *C. rauschi*, *C. reductum*, *C. semerme*, *C. strumosum*, *C. validum*, *C. ventronudum*, *C. villosum*, *C. wegeneri* | Pinnipeds | Arctic fox (*Alopex lagopus*) [*C. wegeneri*]^a^, Domestic dog, Andean fox (*Pseudalopex culpaeus*) [*C. obtuscens*]^b^, Eurasian otter (*Lutra lutra*) [*C. strumosum*]^c^, Laboratory rat (*Rattus novergicus*) [*C. semerme*]^d^, Domestic dog [*C. wegeneri*]^e^, Human [*C.* cf. *validum*]^f^, [*C. villosum*]^g^ |
|  |  |  |
| *C. alaskensis*, *C. cetaceum*, *C. curilensis*, *C. septentrionalis* | Cetaceans |  |
|  |  |  |
| *C. shackletoni*, *C. sudsuche*, *C. turbidum* | Marine birds | Leopard seal (*Hydrurga leptonyx*) [*C. shackletoni*]^h^ |
|  |  |  |
| *C. enhydri* | Sea otter (*Enhydra lutris*) |  |
|  |  |  |
| *C. stanleyi* | Australian water rat (*Hydromys chrysogaster*) |  |
|  |  |  |
| *C. seropedicus* | Domestic dog (*Canis lupus familiaris*) |  |

^a^Two adult (?) worms reported in a single coastal fox as *C. hadweni* [4]

^b^Gravid females reported in natural and experimental infections of stray dogs [5]; 44 adult worms in a single Andean fox [6]

^c^Adult worms reported in 7 out of 56 coastal otters, with intensity ranging from 1 to 28 worms [7]

^d^Gravid worms reported in experimental infections of immunosuppressed rats [8]

^e^Three males and one submature female in 3 out of 339 stray dogs examined [9]

^f^One gravid female from the small intestine from a 70-year-old female [10]

^g^One gravid female from the jejunum from a 73-year-old male [11]

^h^Two gravid females in 1 out of 67 seals examined [12]

**References**

1. Aznar FJ, Pérez-Ponce de León G, Raga JA. Status of *Corynosoma* (Acanthocephala: Polymorphidae) based on anatomical, ecological, and phylogenetic evidence, with the erection of *Pseudocorynosoma*. J Parasitol. 200692:548–564*.*

2. Dunagan TT, Miller DM. Acanthocephalan bibliography. Physiology Department, Southern Illinois University, Carbondale, Illinois, USA, 2008, 853 pp. [Internet].

3. Amin OM. Classification of the Acanthocephala. Folia Parasitol. 2013;60:273–305.

4. Skirnisson K, Eydal M, Gunnarsson E, Hersteinsson P. Parasites of the Arctic fox (*Alopex lagopus*) in Iceland. J Wildl Dis. 1993;29:440–446.

5. Castro M, Martínez R. Proceso del desarrollo de *Corynosoma obtuscens* (Acanthocephala: Polymorphidae) en *Canis familiaris* y su posible implicancia en salud publica. Parasitol Latinoam. 2004;59:26–30.

6. Tantaleán M, Mendoza L, Riofrío F: El zorro Andino, *Pseudalopes culpaeus*, un nuevo huésped para *Corynosoma obtuscens* (Acanthocephala) en el Perú. Rev Peru Biol. 2007;14:51–52.

7. Jefferies DJ, Hanson HM, Harris EA. The prevalence of *Pseudoterranova decipiens* (Nematoda) and *Corynosoma strumosum* (Acanthocephala) in otters *Lutra lutra* from coastal sites in Britain. J Zool. 1990;221:316–321.

8. Valtonen ET, Helle E. Experimental infection of laboratory rats with *Corynosoma semerme* (Acanthocephala). Parasitology. 1982;85:9–19.

9. Kamiya H, Seki N, Tada Y Kamiya M. Some rare helminths from stray dogs of Sapporo. Jap J Vet Res. 1975;23:25–32.

10. Takahashi K, Ito T, Sato T, Goto M, Kawamoto T, Fujinaga A, et al. Infection with fully mature *Corynosoma* cf. *validum* causes ulcers in the human small intestine. J Clin Gastroenterol. 2016;9:114–117.

11. Fujita T, Waga E, Kitaoka K, Imagawa T, Komatsu Y, Takanashi K, et al. Human infection by acanthocephalan parasites belonging to the genus *Corynosoma* found from small bowel endoscopy. Parasitol Int. 2016;65:491–493.

12. Stryukov AA. Invasion of Antarctic phocids seals by acanthocephals. Vestnik Zoologii. 2004;38:23–29 (In Russian).
