# Supplementary material for: From mammals back to birds: Host-switch of the acanthocephalan Corynosoma australe from pinnipeds to the Magellanic penguin Spheniscus magellanicus
Source: PLoS One. 2017 Oct 5;12(10):e0183809. doi: 10.1371/journal.pone.0183809 (PMC5628790; doi:10.1371/journal.pone.0183809)
Supplement: S1 dataset — (DOC) [file pone.0183809.s002.doc]

**S2 Dataset.** List of definitive hosts and localities of *Corynosoma australe* Johnston, 1937.

*Arctocephalus australis* (Zimmermann): Cabo Polonio (34º21′S, 53º5′W) and Isla de Lobos (35º01′S, 54º50′W), Uruguay by Aznar et al. [1, 2]; Claromecó (38º22′S, 60º16′W) and San Clemente del Tuyú (36º30′S, 56º20′W), Argentina by Sardella et al. [3]; northern Patagonia (40º43′–43º20′S, 63º04′–65º07′W), Argentina by Hernández-Orts et al. ([4], present study); Cassino Beach (32º11′S, 52º09′W) and Chui (33º44′S, 53º22′W), Rio Grande do Sul State, Brazil by Silva et al. [5].

*Arctocephalus pusillus* (Schreber): Phillip Island, Australia by Smales [6]; unknown locality by Dailey and Brownell [7].

*Arctocephalus tropicalis* Gray: Cape Town (33º55′S, 18º23′E), South Africa, by Shaughnessy and Ross [8].

*Arctophoca forsteri* (Lesson): New Zealand by Cordes and O’Hara [9].

*Hydrurga leptonyx* (de Blainville): Campbell and Auckland Islands, New Zealand by Johnston and Edmonds [10]; unknown locality by Dailey and Brownell [7]; South Shetlands, Antarctic by Zdzitowiecki [11, 12, 13]; Campbell and Auckland Islands, New Zealand by Smales [6].

*Mirounga leonina* (L.): San Clemente del Tuyú (36º30′S, 56º20′W), Argentina by Sardella et al. [3].

*Neophoca cinerea* (Péron) (*type-host*): Pearson Island, Australia by Johnston [14] (Johnston and Mawson [15] clarified that the sea lion identified as *A. forsteri* in Johnston [14] was actually *N. cinerea*); Pearson Island, Australia by Delyamure [16]; unknown locality by Dailey and Brownell [7]; Pearson and Greenly Islands, Port Adelaide and Dangerous Reef, Australia by Smales [6]; outside the Antarctic by Zdzitowiecki [12, 13].

*Otaria flavescens* (Shaw): Argentina Sea by Morini and Boero [17] (as *C. otariae*); known locality by Dailey and Brownell [7]; outside the Antarctic by Zdzitowiecki [13]; Patagonia, Argentina by Aznar et al. [8]; Puerto Quequén (38º37′S, 58º50′W)., Buenos Aires Province, Argentina by Aznar et al. [2]; northern Patagonia (40º43′–43º20′S, 63º04′–65º07′W), Argentina by Hernández-Orts et al. ([4, 18, 19], present study).

*Phocarctos hookeri* (Gray): Campbell and Auckland Islands, New Zealand by Johnston and Edmonds [10]; Campbell and Auckland Islands, New Zealand by Smales [6]; outside the Antarctic by Zdzitowiecki [12, 13].

*Spheniscusma gellanicus* (Forster): Arraial do Cabo, Rio de Janeiro and Rio Grande do Sul, Brazil by Hernández-Orts et al. (present study).

**References**

1. Aznar FJ, Cappozzo HL, Taddeo D, Montero FE, Raga JA. Recruitment, population structure, and habitat selection of *Corynosoma australe* (Acanthocephala) in South American fur seals, *Arctocephalus australis*, from Uruguay. Can J Zool. 2004;82:741–748.

2. Aznar FJ, Pérez-Ponce de León G, Raga JA: Status of *Corynosoma* (Acanthocephala: Polymorphidae) based on anatomical, ecological, and phylogenetic evidence, with the erection of *Pseudocorynosoma*. J Parasitol. 2006;92:548–564*.*

3. Sardella NH, Mattiucci S, Timi JT, Bastida RO, Rodríguez DH, Nascetti G: *Corynosoma australe* Johnston, 1937 and *C. cetaceum* Johnston & Best, 1942 (Acanthocephala: Polymorphidae) from marine mammals and fishes in Argentinian waters: allozyme markers and taxonomic status. *‎*Syst Parasitol. 2005;61:143–156.

4. Hernández-Orts JS, Montero FE, Juan-García A, García NA, Crespo EA, Raga JA, Aznar FJ: Intestinal helminth fauna of the South American sea lion *Otaria flavescens* and fur seal *Arctocephalus australis* from northern Patagonia, Argentina. J Helminthol. 2013; 87:336–347.

5. Silva RZ, Pereira J.Jr, Cousin JCB: Histological patterns of the intestinal attachment of *Corynosoma australe* (Acanthocephala: Polymorphidae) in *Arctocephalus australis* (Mammalia: Pinnipedia). J Parasit Dis. 2014;38:410–416.

6. Smales LR. Polymorphidae (Acanthocephala) from Australian mammals with descriptions of two new species. Syst Parasitol. 1986;8:91–100.

7. Dailey MD, Brownell Jr, RL. A checklist of marine mammal parasites. In: Ridgway SH, editor. Mammals of the Sea, Biology and Medicine. Springfield: Thomas; 1972. p. 528–589.

8. Shaughnessy PD, Ross GJB. Records of the subantarctic fur seal (*Arctocephalus tropicalis*) from South Africa with notes on its biology and some observations of captive animals. Annls S Afr Mus. 1980;82:71–89.

9. Cordes DO, O’Hara PJ: Diseases of captive marine mammals. NZ Vet J. 1979;27:147–150.

10. Johnston JT, Edmonds SJ: Acanthocephala from Auckland and Campbell islands. Rec Domin Mus. 1953;2:55–61.

11. Zdzitowiecki K. Some antarctic acanthocephalans of the genus *Corynosoma* parasitizing Pinnipedia, with description of three new species. Acta Parasitol. 1984;29:359–377.

12. Zdzitowiecki K. Acanthocephala of the Antarctic. Polish Polar Res. 1986;7:79–117.

13. Zdzitowiecki K. Antarctic Acanthocephala. In: Wägele JW J, Sieg J, editors. Synopses of the Antarctic benthos, Vol. 3. Köenigstein: Koeltz Scientific Books; 1991. p. 1–116.

14. Johnston TH. Entozoa from the Australian hair seal*.* Proc Linn Soc NSW. 1937;62:9–16.

15. Johnston TH, Mawson PM. Nematodes from Australian marine mammals. Rec South Aust Mus. 1941;6:429–434.

16. Delyamure SL. Helminthofauna of marine mammals (ecology and phylogeny). Moscow: Izdatelstvo Akademii Nauk SSSR; 1955 (In Russian).

17. Morini EG, Boero JJ. *Corynosoma otariae* n. sp. (Acanthocephala; Polymorphidae) parásito de un lobo marino (*Otaria flavescens*). Acta Trab Congr Sudam Zoología. La Plata, Argentina; 1959:229–234.

18. Hernández-Orts JS, Timi JT, Raga JA, García-Varela M, Crespo EA, Aznar FJ. Patterns of trunk spine growth in two congeneric species of acanthocephalan: investment in attachment may differ between sexes and species. Parasitology. 2012;7:945–955.

19. Hernández-Orts JS, Smales LR, Pinacho-Pinacho CD, García-Varela M, Presswell B. Novel morphological and molecular data for *Corynosoma hannae* Zdzitowiecki, 1984 (Acanthocephala: Polymorphidae) from teleosts, fish-eating birds and pinnipeds from New Zealand. Parasitol Int. 2017;66:905–916.
